# Arthroscopic removal of palmar intermediate carpal bone fracture fragments in four horses using a transthecal approach through the carpal flexor tendon sheath

**DOI:** 10.1111/vsu.13813

**Published:** 2022-04-11

**Authors:** Charlotte L. Hewitt‐Dedman, Henry D. O'Neill, Bruce M. Bladon

**Affiliations:** ^1^ The University of Edinburgh Royal (Dick) School of Veterinary Studies Edinburgh UK; ^2^ Donnington Grove Equine Hospital Newbury UK

## Abstract

**Objective:**

To describe the removal of palmar intermediate carpal bone (ICB) fracture fragments using a transthecal approach through the carpal flexor tendon sheath.

**Study design:**

Case series.

**Animals:**

Four horses with fractures of the palmar aspect of the ICB.

**Methods:**

Two horses were injured when falling and two during recovery from general anesthesia. Three horses underwent MRI to supplement conventional imaging. Three horses had concurrent fractures of the radial and/or accessory carpal bones. Conventional proximolateral carpal sheath arthroscope and instrument portals were used, supplemented with a medial instrument portal through the carpal flexor retinaculum to access the palmar carpal ligament. Optimized dissection through the latter was facilitated by needle guidance and radiography. The fragment was dissected from the soft tissue attachments and the palmar ICB fragments retrieved through the carpal sheath in all horses. Surgery time was 85 to 142 min.

**Results:**

Limitations of this technique include a long surgery time and the potential for hemorrhage to impair visibility during surgery. All four horses were discharged 3 to 8 days postoperatively. Three horses returned to full athletic work within 9 months postoperatively and one horse was euthanized due to persistent lameness.

**Conclusion:**

A tenoscopic transthecal carpal flexor tendon sheath approach provides access for removal of palmar ICB fracture fragments but should be viewed as an advanced arthroscopic procedure.

**Clinical significance:**

A transthecal approach through the carpal flexor tendon sheath offers an alternative technique for removal of palmar ICB fracture fragments.

## 1 INTRODUCTION

Palmar fractures of the carpal bones are a recognized cause of lameness, often associated with trauma during recovery from general anesthesia.^1^, ^2^ Involvement of the palmar intermediate carpal bone (ICB) is, however, infrequently reported, occurring either alone or in combination with other fractures.^1^, ^2^

To the authors’ knowledge, no studies exist that specifically assess outcome following palmar ICB fractures. In one case series, conservative management of palmar radial carpal bone fractures with or without other palmar carpal bone fractures was reported to have a poor prognosis.^1^ All four horses treated conservatively remained chronically lame and three were euthanized. Improved prognosis was reported following surgical fragment removal, with three out of six returning to some level of exercise.^1^ A study of racehorses with palmar osteochondral fragments of the radial or third carpal bones found that horses undergoing fragment removal had more starts and higher earnings post‐injury than conservatively managed horses.^3^

Arthroscopic approaches to the medial and lateral aspects of the palmar antebrachiocarpal joint have previously been described, however, portals are in close proximity compared to the dorsal aspect of the joint making triangulation more challenging.^2^, ^4^ Some ICB fragments can be retrieved using the palmaromedial approach to the antebrachiocarpal joint.^2^, ^4^ Other studies found arthroscopic access to the palmar ICB to be limited using these approaches, particularly to view the palmaroproximal ICB.^1^, ^4^ Thus, many ICB fracture fragments remain inaccessible using this approach.^2^ One report describes the removal of a large fragment from the palmar aspect of the ICB using a combined open tenotomy and arthrotomy approach via the carpal flexor tendon sheath. This horse returned to full work 20 months following surgery.^5^

The carpal flexor tendon sheath (*vagina synovialis communis mm. flexorum*) runs in close proximity to the palmar aspect of the carpal bones, starting proximal to the antebrachiocarpal joint and extending distally to the location where the deep digital flexor tendon (DDFT) merges with its accessory ligament.^6^ Tenoscopic approaches have been described for removal of comminuted accessory carpal bone (ACB) fractures, radial osteochondromas, radial physeal exostoses and release of the accessory ligaments of the superficial and deep digital flexor tendons.^7^, ^8^, ^9^, ^10^, ^11^, ^12^, ^13^ The larger capacity of the carpal flexor tendon sheath may afford greater flexibility for instrument portal placement than the palmar antebrachiocarpal joint.^2^, ^9^

The aim of this study was to describe the surgical technique and outcome of removal of palmar ICB fracture fragments using a transthecal approach through the carpal flexor tendon sheath.

## 2 MATERIALS & METHODS

### 2.1 Case selection

Horses undergoing arthroscopic palmar ICB fracture fragment removal using a transthecal approach through the carpal sheath of the digital flexor tendons, with or without removal of concurrent palmar fractures of other carpal bones at a single referral hospital between August 2017 and October 2020 were included.

### 2.2 Signalment and presentation

Four horses consisting of two geldings, one mare and one filly, with ages ranging from 3 to 13 years were included (Table 1). Breeds included three Thoroughbreds and one Irish Sport Horse. Two horses were racehorses in training, one was used for show jumping and one was a leisure horse. Fractures occurred due to a fall during exercise (*n* = 2) or anesthetic recovery *(n* = 2). On presentation, all horses showed between a grade 2 and grade 3 (AAEP scale) lameness at the trot with pain on carpal flexion, two horses showed effusion of the antebrachiocarpal joint and 1 showed swelling at the palmarolateral aspect of the carpus.

**TABLE 1:** Case overview of four horses that underwent removal of palmar intermediate carpal bone (ICB) fracture fragments using a tenoscopic approach through the carpal sheath of the flexor tendons

Variables	Horse 1	Horse 2	Horse 3	Horse 4
Signalment				
Age (years)	11	13	7	3
Sex	Mare	Gelding	Gelding	Filly
Breed	TB	Irish Sport Horse	TB	TB
History				
Etiology	Fell onto right carpus whilst jumping	Anesthetic recovery – colic surgery	Anesthetic recovery – right fore digital flexor tendon sheath tenoscopy	Fell during race training
Affected limb	Right fore	Right fore	Right fore	Left fore
Time between injury and presentation	4 weeks	4 weeks	4 weeks	1 day
Diagnostic imaging				
Radiography	Yes	Yes	Yes	Yes
Ultrasonography	No	Yes	No	No
MRI	Yes	Yes	No	Yes
Diagnosis	Simple, complete, displaced, articular fracture of the palmar ICB	Simple, complete, displaced, articular fracture of the palmar ICB	Simple, complete, displaced, articular fracture of the palmar ICB	Simple, complete, displaced, articular fracture of the palmar ICB
			Comminuted, complete, mildly displaced, articular fracture of the accessory carpal bone (ACB)	Comminuted, complete, markedly displaced, nonarticular, frontal plane fracture of the ACB
		Simple, complete, displaced, articular fracture of the palmar radial carpal bone		
		Mild osteophyte formation at the dorsodistal aspect of the radius and the dorsal aspect of the radial and IC bones		
Surgery				
Procedure	Right carpal sheath tenoscopy	Right carpal sheath tenoscopy	Right carpal sheath tenoscopy	Left carpal sheath tenoscopy
			Right antebrachiocarpal joint arthroscopy	
		Right antebrachiocarpal joint arthroscopy		
		Right middle carpal joint arthroscopy		
Surgery time (min)	85	145	141	142
Intraoperative Complications	None	None	Hemorrhage obscuring visibility	Inadvertent entry of left antebrachiocarpal joint
			Second surgery 3 days later	
Postoperative complications	None	None	None	Post‐anesthetic myopathy
Follow‐up time (months)	30	4	15	8
Outcome	Returned to previous levels of work by 10 months post‐surgery	Euthanasia at 4 months post‐surgery	Antebrachiocarpal joint medicated with 10 mg triamcinolone acetonide at 24 weeks post‐surgery	Antebrachiocarpal joint and carpal sheath medicated with 10 mg triamcinolone acetonide in each at 16 weeks post‐surgery
	Remained in full work at 30 months post‐surgery			
			Back in training by 9 months post‐surgery	
				Back in pretraining by 4 months post‐surgery
			First race at 15 months post‐surgery	
				Returned to full training at 6 months post‐surgery
				First race at 8 months post‐surgery

### 2.3 Imaging

**FIGURE 1:**
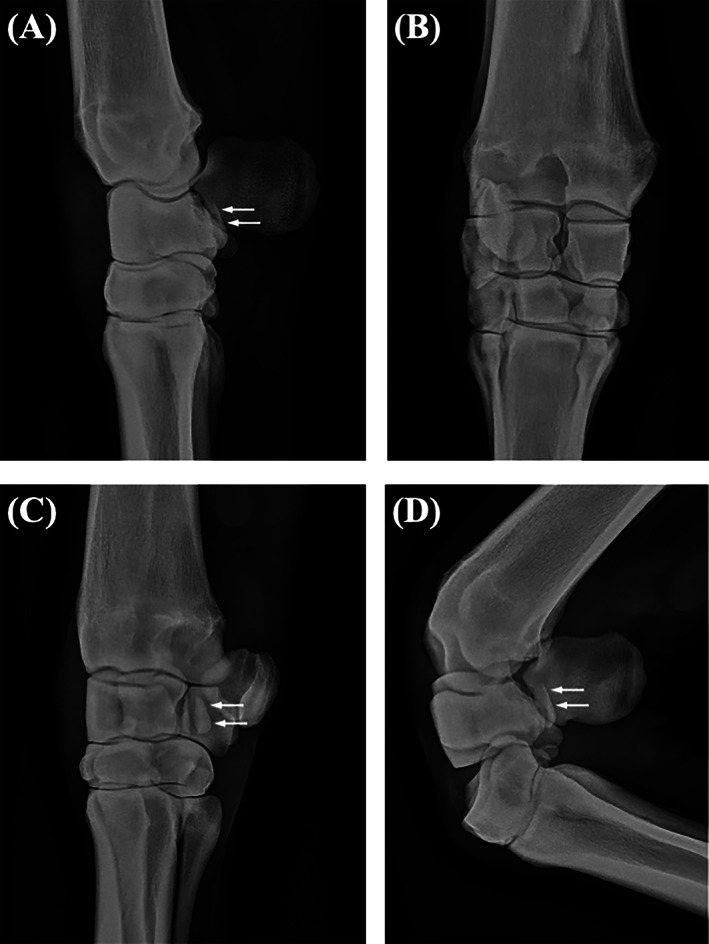
(A) Lateromedial, (B) dorsopalmar, (C) dorsolateral‐palmaromedial oblique and (D) flexed lateromedial radiographic views of the right carpus of Horse 1 showing a simple, complete, displaced, articular fracture of the palmar aspect of the intermediate carpal bone (arrows)

Horses underwent radiographic examination, which revealed a simple, complete, displaced, articular fracture of the palmar aspect of the ICB (Figure 1). Horse 2 also had a simple, complete, displaced, articular fracture of the palmar aspect of the radial carpal bone. Horses 3 and 4 also had comminuted, complete, displaced frontal plane fractures of the ACB. Horse 2 underwent ultrasonographic examination which confirmed the position of the fracture fragments and identified small areas of mineralized debris in the dorsal aspect of the antebrachiocarpal joint.

**FIGURE 2:**
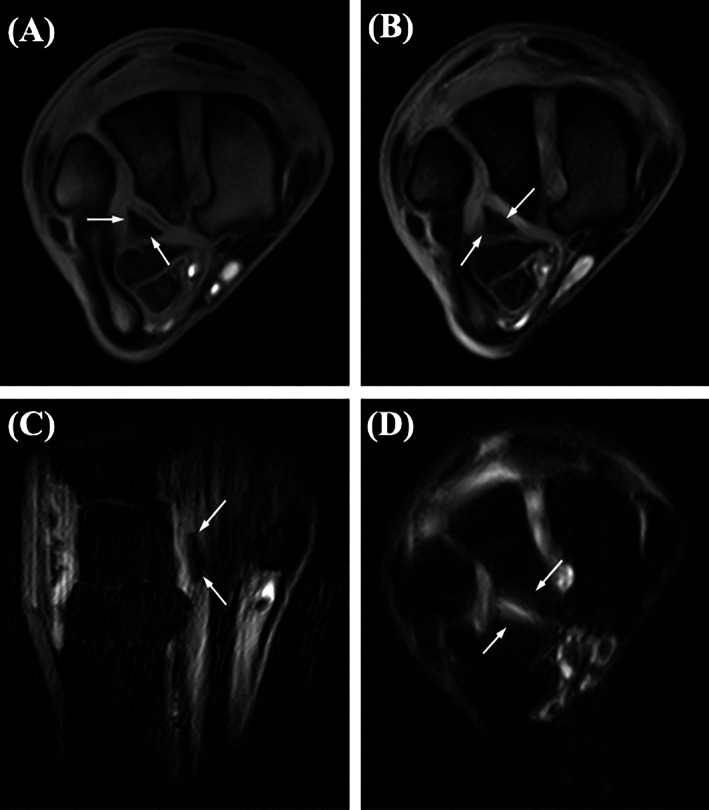
(A) T1 and (B) T2* weighted gradient echo transverse magnetic resonance sequences of the right carpus of Horse 1 showing an area of hypointensity palmar to the intermediate carpal bone (ICB) (arrows) consistent with a fracture fragment from the ICB. (C) Sagittal and (D) transverse short tau inversion recovery (STIR) sequences of the right carpus of Horse 1 showing the ICB fracture fragment (arrows (C)) with an area of fluid signal in the ICB (arrows (D))

Horses 1, 2 and 4 underwent standing MRI of the affected carpus using a 0.27 Tesla open coil permanent magnet (Hallmarq Veterinary Imaging, Guilford, UK). T1‐weighted and T2*‐weighted gradient echo and short tau inversion recovery (STIR) fast spin echo sequences were acquired of the antebrachiocarpal joint in the transverse, sagittal and frontal planes. All cases showed an area of hypointensity palmar to the ICB on T1 and T2*‐weighted sequences consistent with a fracture fragment. This was associated with focal hyperintensity on STIR sequences (Figure 2) and hyperintensity with a surrounding hypointense zone on T2* sequences within the palmar ICB. This was interpreted as a “high water” or “high fluid” signal with a fluid fat phase cancellation artifact on T2* sequences, a finding colloquially known as bone edema.^14^, ^15^

### 2.4 Surgical technique

Procaine penicillin (22 000 IU/kg IM), gentamicin (6.6 mg/kg IV) and phenylbutazone (4.4 mg/kg IV) were administered 30–60 minutes prior to induction. Following premedication with acepromazine (0.03 mg/kg IV) and medetomidine (7 μg/kg IV), general anesthesia was induced with ketamine (2.2 mg/kg IV) and diazepam (0.05 mg/kg IV), maintained with isoflurane and a continuous rate infusion of medetomidine (3.5 μg/kg/h). Morphine (0.1 mg/kg IM) was administered upon completion of surgery.

**FIGURE 3:**
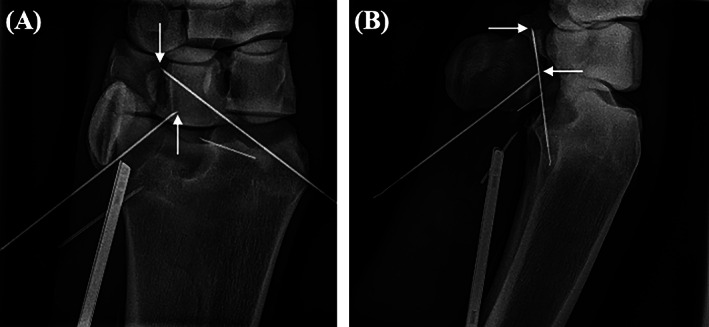
Dorsopalmar (A) and lateromedial (B) radiographs of the right carpus of Horse 1 taken intraoperatively showing two 3.5 inch spinal needles placed to mark the proximal and distal border (white arrows) of the intermediate carpal bone fragment. The arthroscope can be visualized on the lateral aspect of the image and the two 1.5 inch needles present had been placed into the medial and lateral aspect of the sheath for egress prior to instrument portal placement

**FIGURE 4:**
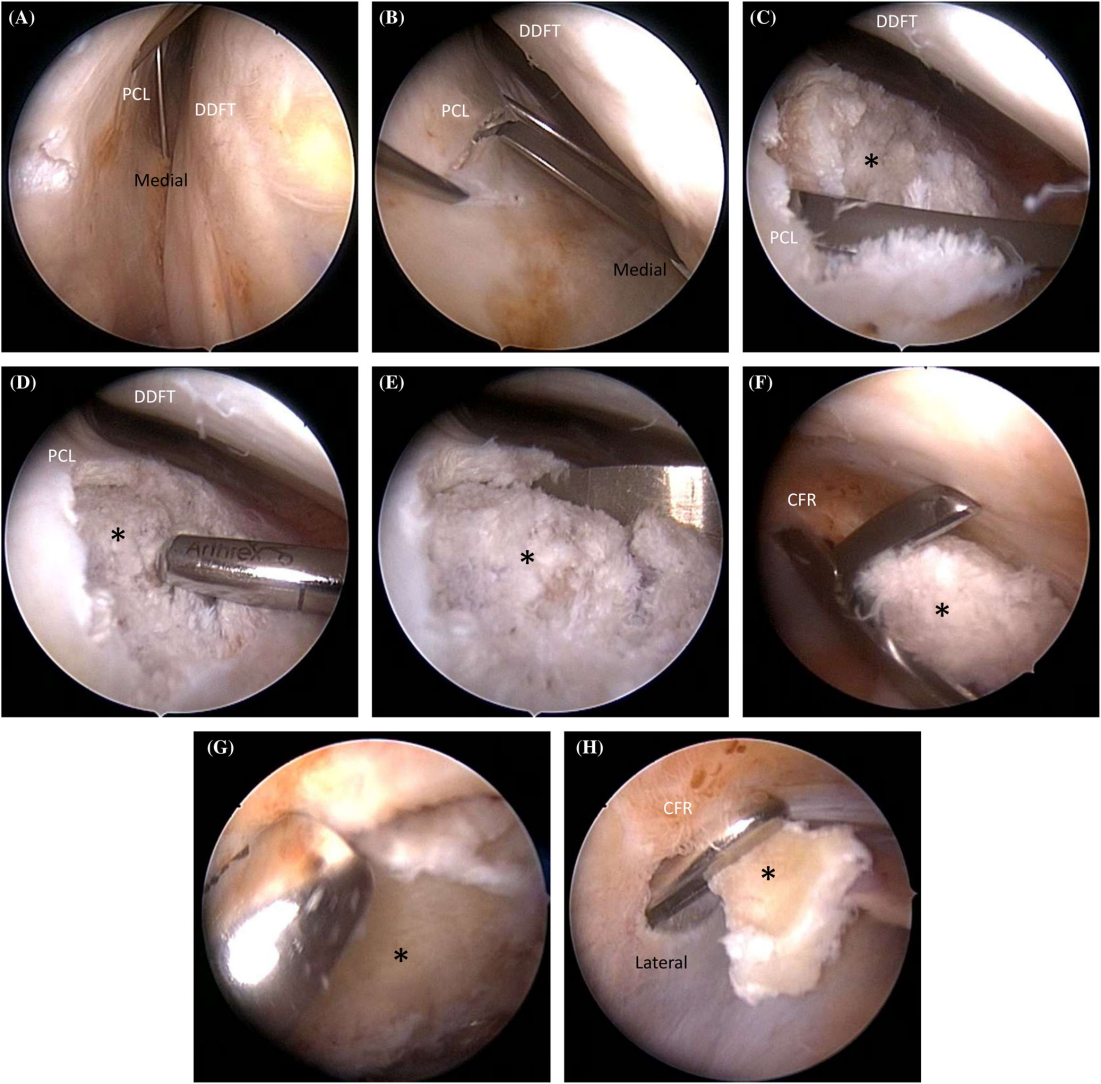
A series of images captured during tenoscopy of the right carpal sheath and removal of the palmar intermediate carpal bone fragment (*). The following structures can be visualized: palmar carpal ligament (PCL), deep digital flexor tendon (DDFT), carpal flexor retinaculum (CFR). (A) Needle placement under arthroscopic visualization. (B) An incision into the PCL using a 60° hoe blade through a medial instrument portal. (C–E) Continued dissection of the area using the hoe blade (C), a motorized soft tissue synovial resector (D) and the periosteal elevator (E). (F) Attempted fragment removal using Ferris‐Smith rongeurs. (G) Fragment debridement using a motorized bone blade. (H) Removal of the debrided fracture fragment through the lateral instrument portal

**FIGURE 5:**
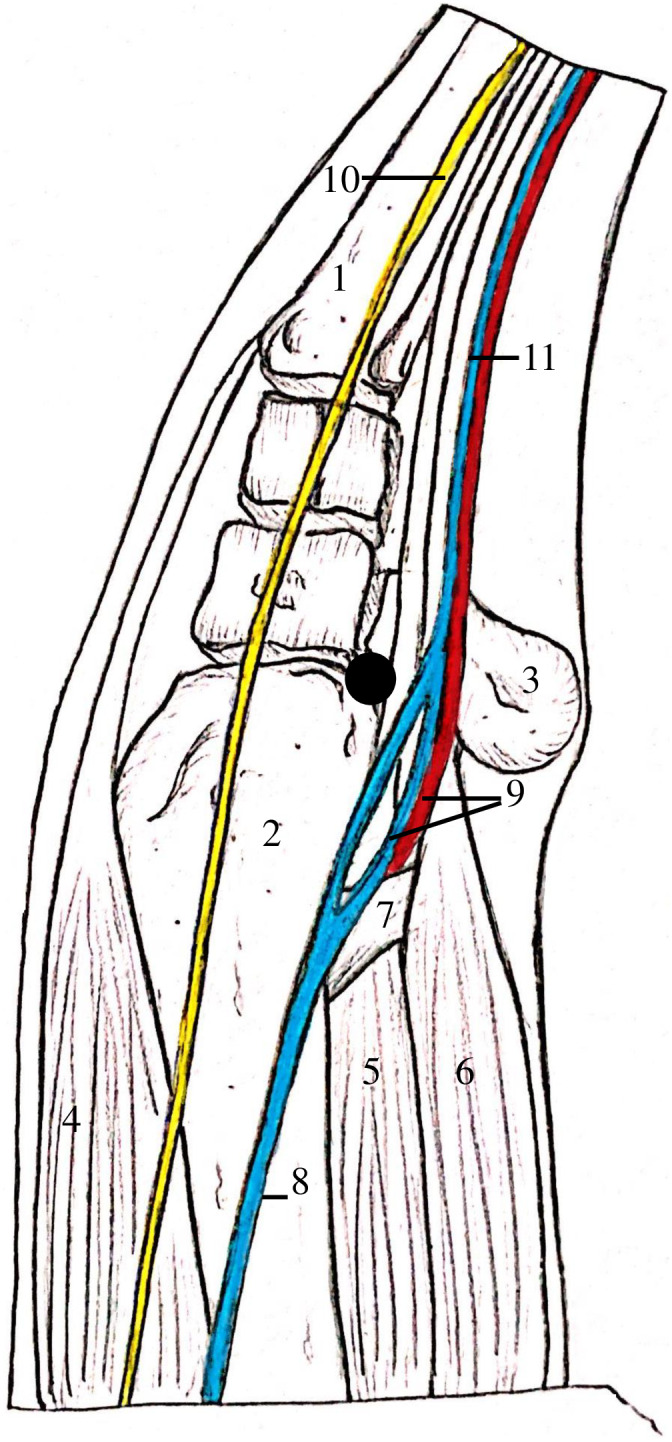
Anatomy of the medial aspect of the carpal sheath simulating a patient in dorsal recumbency. The superficial neurovascular structures have been superimposed over the radius and carpal bones. The medial portal position immediately caudal to the radius is indicated (black circle). (1) Metacarpus II, (2) radius, (3) accessory carpal bone, (4) extensor carpi radialis, (5) deep digital flexor muscle, (6) superficial digital flexor muscle (7) superior check ligament, (8) cephalic vein, (9) median artery and vein, (10) medial cutaneous nerve of forearm and (11) medial palmar vein

All horses were positioned in dorsal recumbency with the limb supported in approximately 20° of carpal flexion. An 18‐gauge 1.5‐inch needle was placed into the proximolateral aspect of the carpal flexor tendon sheath followed by distension with sterile polyionic fluid (Vetivex 11, Hartmann's Solution). An arthroscope portal was made at the point of maximal distension proximal to the radial physis as previously described.^11^, ^12^ The site for the lateral instrument portal was determined by placing an 18‐gauge needle into the sheath immediately proximal to the ACB. Following tenoscopic assessment of the sheath the proximal and distal limits of the fragment were marked using two 18‐gauge spinal needles, with the bevel embedded into the palmar carpal ligament (PCL). Orthogonal radiographs confirmed accurate placement. This required a medial approach to the sheath with a spinal needle to mark the distal margin of the fragment. (Figures 3 and 4A). An additional medial instrument portal was created level with the distal needle, through the carpal flexor retinaculum and immediately caudal to the radius thus avoiding iatrogenic trauma to the radial vein and artery (Figure 5; Video S1).^16^ Using a 4 mm Arthro‐Lok rosette blade and a 4 mm Arthro‐Lok hoe blade angled at 60° (Beaver‐Visitec International, Waltham, Massachusetts), the blade was introduced through the medial portal and an incision was made into the PCL between the two needles using tenoscopic visualization and guidance (Figure 4B and Video S1). Generally, this incision was ~1 cm long and was enlarged to ~5 mm width (Video S1). Continued dissection of the area was performed using a combination of the Arthro‐Lok blade, a McIlwraith periosteal elevator (Sontec Instruments, Cenntenial, Colorado), a suction punch instrument (Dyonics, Smith and Nephew Endoscopy, UK) and a motorized soft tissue synovial resector (Saber 3.8 mm x 13 cm blade; Arthrex Vet Systems, Germany) (Figure 4C–E and Video S1). Instruments were alternated between the medial and lateral portals to facilitate complete dissection of the area. The fragments all had extensive soft tissue attachments which required dissection. The fragment was then grasped and withdrawn in toto into the carpal sheath and subsequently exiting the body through the skin portal (Figure 4H and Video S1). In one case, the 4 x 10 mm Ferris Smith rongeurs (Sontec Instruments, Cenntenial, Colorado) were unable to completely engage the jaws around the fragment, which was lowered into the proximal recess of the sheath and progressively debulked using a motorized bone burr (ClearCut Round, 8 Flute, 5.5 mm x 13 cm; Arthrex Vet Systems, Germany) prior to removal (Figure 4G, H and Video S1). The fracture bed and surrounding PCL was debrided of any debris using the motorized synovial resector. Intraoperative radiographs confirmed complete removal of the fragment. The sheath was lavaged extensively and the portals were closed using simple interrupted sutures of 3‐0 USP polypropylene or nylon. A pressage bandage was placed prior to recovery from general anesthesia.

### 2.5 Intraoperative findings

All horses underwent carpal sheath tenoscopy as described. Although a standard lateral instrument portal was created in all cases, direct access to the PCL and fragment was best achieved with a medial instrument portal due to the obstruction created by the ACB on the corresponding lateral aspect of the limb. All palmar ICB fracture fragments were successfully retrieved through the carpal sheath.

Horse 2 initially underwent arthroscopy of the dorsal and palmar pouches of the antebrachiocarpal joint to remove a radial carpal bone fragment. This was followed by tenoscopy of the carpal sheath to remove a palmar ICB fracture fragment and finally diagnostic arthroscopy of the middle carpal joint which revealed a full thickness cartilage erosion on the dorsodistal radial carpal bone.

Horse 3 had a concurrent fracture of the ACB and underwent two procedures under general anesthesia 3 days apart. During the first procedure, a fracture fragment from the palmar ICB was initially retrieved through the carpal sheath and three fragments were then removed from the ACB through the palmar aspect of the antebrachiocarpal joint. Intraoperative hemorrhage obscured the visibility towards the end of the first surgery, therefore, the procedure was stopped, and the horse recovered from anesthesia. Radiographs 24 h later revealed a large fragment off the proximal ACB remained. Two days later, the horse was anesthetized and this fragment was successfully removed through the palmar antebrachiocarpal joint.

The initial arthroscope portal entered the palmar aspect of the antebrachiocarpal joint in Horse 4. Another portal was created, allowing entry into the carpal sheath.

## 3 RESULTS

### 3.1 Surgical outcome

Removal of palmar ICB fracture fragments using a transthecal approach through the carpal flexor tendon sheath was attempted in four horses and successful in all cases. Horse 3 required a second procedure to remove a remaining ACB fracture fragment. Mean surgery time was 128 min (range: 85–142 min) and mean anesthesia time was 179 min (range: 131–201 min).

### 3.2 Postoperative management

Horse 4 which had an anesthetic time of 182 min and a surgery time of 142 min experienced a post‐anesthetic myopathy which resolved within 3 days following treatment with flunixin meglumine (1.1 mg/kg BID) and 24 h of intravenous fluid therapy. Postoperative radiographs were taken of all four horses on the day following surgery to confirm removal of all fracture fragments. Horses received a 3‐to‐5‐day course of procaine penicillin (22 000 IU/kg IM BID) and gentamicin (6.6 mg/kg IV SID). Oral phenylbutazone (2.2 mg/kg BID) was continued for 5 to 14 days. Horses remained in the hospital for 3 to 8 days postoperatively (median = 6 days). Six weeks of box rest was recommended followed by 2 months of horse walker exercise and a further 3 months of paddock rest.

### 3.3 Follow‐up

All horses were discharged from the hospital. Suture removal was performed 2 weeks postoperatively and no complications were reported. Follow‐up information was collected from owners, trainers or referring veterinary surgeons at a minimum of 8 months post‐diagnosis.

Horse 1 was re‐examined at 12 weeks post‐surgery and was sound at the trot with good carpal flexion. Radiographic examination of the right carpus at this time revealed no significant findings. This horse had returned to previous levels of athletic work by 9 months and remained in full work at 30 months postoperatively.

Horse 2 was re‐examined at 17 weeks post‐surgery. The horse remained right forelimb lame and was painful to flexion of the carpus. Radiographic examination of the right carpus at this time revealed several osteophytes at the level of the dorsodistal radius with irregularity of the distal radius and dorsal aspect of the radial carpal bone. These findings were consistent with marked antebrachiocarpal joint osteoarthritis. Given these findings, this horse was euthanized at the owner's request.

Horse 3 was re‐examined at 10 weeks post‐surgery and was sound at the trot with good carpal flexion. Radiographic examination of the right carpus at 24 weeks post‐surgery revealed a small osteophyte on the dorsolateral aspect of the distal radius with mild irregularity of the dorsal aspect of the radial carpal bone. This was consistent with mild osteoarthritis of the antebrachiocarpal joint. The right antebrachiocarpal joint was medicated with 10 mg triamcinolone acetonide at 24 weeks post‐surgery. This horse had returned to training by 9 months and first raced at 15 months post‐operatively.

Horse 4 was re‐examined at 6 weeks post‐surgery. The horse was lame on the contralateral limb and was diagnosed with a subsolar abscess. Radiographic examination of the left carpus at 6 weeks revealed no significant findings. Re‐examination at 12 weeks post‐surgery revealed that the horse was sound at the trot. The left antebrachiocarpal joint and carpal sheath were medicated with 10 mg triamcinolone acetonide in each at 16 weeks post‐operatively. This horse was sound and had returned to pre‐training by 4 months, full training by 6 months and first raced at 8 months post‐surgery.

## 4 DISCUSSION

This report provides a description of a novel tenoscopic transthecal carpal flexor tendon sheath approach for palmar ICB fracture fragment removal. Fracture fragments were successfully removed in all 4 horses. Although an open technique through the carpal sheath has previously been described where the horse returned to work at 20 months post‐surgery,^5^ minimally invasive techniques are now preferred as they allow for better visualization, reduced soft tissue trauma and a quicker return to exercise.^17^, ^18^ Access to ICB fragments with an arthroscopic approach to the palmaromedial antebrachiocarpal joint can be limited by the working space within this synovial joint, and the size and location of the fragments in the cases presented in this report may have precluded this approach for removal.^1^, ^4^ While the tenoscopic approach used was successful, it is not without risks. Tenoscopy of the carpal sheath is reported to have a higher risk of synovial sepsis, and intraoperative hemorrhage may result in failure to remove the fragments and the need for a second procedure.^19^

The standard proximolateral approach to the carpal sheath provides good visualization of the PCL on the dorsal aspect of the sheath as well as allowing good visualization of the DDFT.^11^, ^12^A medial tenoscopic portal is seldom used, as medial distension is limited by the carpal flexor retinaculum and multiple neurovascular structures overlying the medial carpal sheath. At the proximal end of the carpal sheath, the median vein, artery and nerve run superficial to the medial border of the sheath. Further distally the carpal flexor retinaculum contains the radial artery and vein whilst the medial palmar artery and nerve are contained within the common mesotenon of the superficial digital flexor tendon (SDFT) and the DDFT. Contrary to this, the lateral approach is not impeded by the presence of any neurovascular structures.^6^, ^9^, ^12^

A medial approach to remove a radial osteochondroma in a single horse has been described with no reported complications.^20^ In this report, the horse was positioned in lateral recumbency with the affected leg down and both the arthroscope portal and the instrument portal were created on the medial aspect of the limb. This differs from the current case series where, debridement and removal of the fragment was initially attempted through a lateral instrument portal proximal to the ACB. However, when positioning the needles to outline the proximal and distal limits of the fragment, it was found that a medial portal was required. Given the medial neurovascular structures described, the surgeon must combine careful palpation with a skin approach immediately caudal to the radius to avoid iatrogenic vascular trauma during medial portal placement. In this study hemorrhage was reported to limit visibility in one case; this was noted following significant soft tissue dissection within the sheath rather than at the point of initial portal placement.

Accessory carpal bone fragments can be visualized within the sheath, requiring minimal soft tissue dissection.^13^ In contrast palmar ICB fragments are dorsal to the thick PCL and cannot be visualized within the carpal sheath. Therefore, sharp dissection through the PCL using radiographic guidance was necessary. The PCL forms the dorsal wall of the carpal canal, separating the carpal sheath from the carpal joints.^9^, ^12^ Two case reports describe a poor prognosis following PCL rupture whilst another reports a good prognosis following a partial tear of the ligament.^21^, ^22^, ^23^ None of the horses in the current report showed evidence of carpal hyperextension on postoperative radiographs suggesting that the PCL was still intact. A good outcome was reported in three horses, therefore it seems that the incision through the PCL is well tolerated.

Three horses in this study returned to previous levels of athletic work whilst one horse was euthanized. The euthanized horse showed evidence of antebrachiocarpal joint osteoarthritis on preoperative radiographs, whilst this was not the case in the other horses. Although two of the other horses had concurrent ACB fractures, the euthanized horse was the only horse with a concurrent palmar radial carpal bone fracture fragment. Both of these factors may have contributed to the negative outcome in this case.

The outcome of the described technique is comparable with a previous case series where three out of six horses returned to exercise following removal of palmar carpal bone fracture fragments, although not specifically ICB,^1^ and a good outcome was reported following removal of an ICB fragment from one horse via an open tenovaginotomy.^5^ In 31 racehorses with palmar carpal osteochondral fragments of the radial or third carpal bones, 51.6% of horses returned to racing following arthroscopic removal. However, many of these horses had multiple fragments and all showed some evidence of articular cartilage damage.^3^

This tenoscopic transthecal carpal flexor tendon sheath approach for removal of palmar ICB fracture fragments has potential complications. Firstly, the authors consider this an advanced arthroscopic procedure with familiarity in carpal sheath anatomy and precise soft tissue dissection a prerequisite. Post‐anesthetic myopathy occurred in one horse in the current study and has been associated with a prolonged duration of anesthesia, hypotension, poor padding or positioning of the horse during anesthesia.^24^, ^25^, ^26^ In this case series, three out of four horses had concurrent fractures of other carpal bones requiring removal of multiple fragments and in two horses, arthroscopy of other joints was required. As expected, the horse with a simple, complete, displaced, articular fracture of the palmar aspect of the ICB without concurrent pathology had the shortest surgery time of 85 min. Hemorrhage obscured visibility in one case. This is often reported during desmotomy of the accessory ligament of the SDFT.^9^ In many cases the combination of positioning the horse in dorsal recumbency and the fluid pressure is sufficient to prevent hemorrhage. The use of bipolar laparoscopic cautery forceps or a hemostat could be considered if the bleeding can be localized.^9^ Alternatively, postponing further surgery until a few days later may be a better option.

A high incidence of postoperative osteoarthritis was noted in this case series despite fragment removal, with two horses showing evidence of antebrachiocarpal joint osteoarthritis on postoperative radiographs. A similar finding was reported in a previous study with two out of 10 horses subjected to euthanasia at 2‐ and 9‐months post‐surgery due to severe osteoarthritis. This was suggested to be due to delay in fragment removal.^1^ In the current series there was a delay of 4 weeks between injury and diagnosis for three horses including both horses that developed osteoarthritis. Additionally, the presence of osteoarthritis both on preoperative radiographs and during arthroscopy may explain the severity of osteoarthritis postoperatively in the case of Horse 2. These findings support early diagnosis and intervention as palmar carpal bone fractures can be a career‐limiting condition.^1^

Based on this short case series, a tenoscopic transthecal carpal flexor tendon sheath approach provides access for removal of palmar ICB fracture fragments, allowing a quicker return to full work than fragment removal via an open approach. Complications may be avoided by minimizing soft tissue dissection and by appropriate positioning and sufficient padding of the horse during surgery. Every effort should be made to keep anesthesia time to a minimum and a staged removal of fragments may be required in more complex cases. Intra‐articular medication with corticosteroids may be a useful adjunctive therapy in the management of postoperative osteoarthritis. A larger series is required to accurately determine the prognosis for this technique.

## CONFLICT OF INTEREST

The authors declare no conflicts of interest related to this study.

## Supporting information


**Video S1.** Tenoscopic removal of an intermediate carpal bone fragment from the carpal flexor tendon sheath

## References

[1] Wilke M , Nixon AJ , Fau‐Malark J , Malark J , Fau‐Myhre G , Myhre G . Fractures of the palmar aspect of the carpal bones in horses: 10 cases (1984‐2000). J Am Vet Med Assoc. 2001;219(6):801‐804. 11561657 10.2460/javma.2001.219.801

[2] McIlwraith CW , Nixon AJ , Wright IM . Chapter 4 ‐ diagnostic and surgical arthroscopy of the carpal joints. In: McIlwraith CW , Nixon AJ , Wright IM , eds. Diagnostic and Surgical Arthroscopy in the Horse (Fourth Edition). Mosby; 2015:45‐110.

[3] Getman LM , Southwood LL , Fau‐Richardson DW , Richardson DW . Palmar carpal osteochondral fragments in racehorses: 31 cases (1994‐2004). J Am Vet Med Assoc. 2006;228(10):1551‐1558. 16677125 10.2460/javma.228.10.1551

[4] Cheetham J , Nixon AJ . Arthroscopic approaches to the palmar aspect of the equine carpus. Vet Surg. 2006;35(3):227‐231. 16635001 10.1111/j.1532-950X.2006.00141.x

[5] Dabareiner RM , Sullins KE , Bradley W . Removal of a fracture fragment from the palmar aspect of the intermediate carpal bone in a horse. J Am Vet Med Assoc. 1993;203(4):553‐555. 8407515

[6] Leach D , Harland R , Burko B . The anatomy of the carpal tendon sheath of the horse. J Anat. 1981;133(Pt 2):301‐307. 7333955 PMC1167672

[7] Southwood LL , Stashak TS , Fehr JE , Ray C . Lateral approach for endoscopic removal of solitary osteochondromas from the distal radial metaphysis in three horses. J Am Vet Med Assoc. 1997;210(8):1166‐1168. 9108924

[8] Wright IM , Minshall GJ . Clinical, radiological and ultrasonographic features, treatment and outcome in 22 horses with caudal distal radial osteochondromata. Equine Vet J. 2012;44(3):319‐324. 21848535 10.1111/j.2042-3306.2011.00438.x

[9] McIlwraith CW , Nixon AJ , Wright IM . Chapter 12 ‐ Tenoscopy. In: McIlwraith CW , Nixon AJ , Wright IM , eds. Diagnostic and Surgical Arthroscopy in the Horse (Fourth Edition). Mosby; 2015:344‐386.

[10] Southwood LL , Stashak TS , Kainer RA , Wrigley RH . Desmotomy of the accessory ligament of the superficial digital flexor tendon in the horse with use of a tenoscopic approach to the carpal sheath. Vet Surg. 1999;28(2):99‐105. 10100763 10.1053/jvet.1999.0099

[11] Cauvin ERJ , Munroe GA , Boyd JS . Endoscopic examination of the carpal flexor tendon sheath in horses. Equine Vet J. 1997;29(6):459‐466. 9413719 10.1111/j.2042-3306.1997.tb03159.x

[12] Southwood LL , Stashak TS , Kainer RA . Tenoscopic anatomy of the equine carpal flexor synovial sheath. Vet Surg. 1998;27(2):150‐157. 9525031 10.1111/j.1532-950x.1998.tb00112.x

[13] Minshall GJ , Wright IM . Frontal plane fractures of the accessory carpal bone and implications for the carpal sheath of the digital flexor tendons. Equine Vet J. 2014;46(5):579‐584. 24164449 10.1111/evj.12203

[14] Dyson S , Murray R , Schramme M , Branch M . Lameness in 46 horses associated with deep digital flexor tendonitis in the digit: diagnosis confirmed with magnetic resonance imaging. Equine Vet J. 2003;35(7):681‐690. 14649360 10.2746/042516403775696294

[15] Dyson SJ , Murray R , Schramme MC . Lameness associated with foot pain: results of magnetic resonance imaging in 199 horses (January 2001‐December 2003) and response to treatment. Equine Vet J. 2005;37(2):113‐121. 15779622 10.2746/0425164054223804

[16] Probst A , Macher R , Fau‐Hinterhofer C , et al. Anatomical features of the carpal flexor retinaculum of the horse. *Anatomia Histologia* . Embryologia. 2008;37:415‐417. 10.1111/j.1439-0264.2008.00867.x 18513274

[17] McIlwraith CW , Nixon AJ , Wright IM . Chapter 3 ‐ general technique and diagnostic arthroscopy. In: McIlwraith CW , Nixon AJ , Wright IM , eds. Diagnostic and Surgical Arthroscopy in the Horse (Fourth Edition). Mosby; 2015:28‐44.

[18] Frisbie DD , Johnson SA . Chapter 81 ‐ surgical treatment of joint disease. In: Auer JA , Stick JA , Kümmerle JM , Prange T , Saunders WB , eds. Equine Surgery. Fifth ed.; Missouri: Elsevier; 2019:1363‐1373.

[19] Hawthorn A , Reardon R , O'Meara B , James F , Bladon B . Post operative synovial sepsis following endoscopic surgery: increased risk associated with the carpal sheath. Equine Vet J. 2016;48(4):430‐433. 26095237 10.1111/evj.12472

[20] Squire KR , Adams SB , Widmer WR , Coatney RW , Habig C . Arthroscopic removal of a palmar radial osteochondroma causing carpal canal syndrome in a horse. J Am Vet Med Assoc. 1992;201(8):1216‐1218. 1429162

[21] Wright IM . Ligaments associated with joints. Sl: Maththew R Rantanen; 1996:

[22] Pepe M , Beccati F , Gialletti R , Moriconi F . Bilateral rupture of the palmar carpal ligament in a horse suffering from acute diaphragmatic hernia. J Equine Vet. 2013;33(1):57‐61.

[23] Barba M , McMaster M , Albanese V , Cole R , Caldwell F , Schumacher J . Carpal hyperextension in a Percheron mare caused by a palmar carpal ligament tear. Equine Vet Educ. 2014;26(7):347‐352.

[24] Young SS , Taylor PM . Factors influencing the outcome of equine anaesthesia: a review of 1,314 cases. Equine Vet J. 1993;25(2):147‐151. 8467775 10.1111/j.2042-3306.1993.tb02926.x

[25] Franci P , Leece EA , Brearley JC . Post anaesthetic myopathy/neuropathy in horses undergoing magnetic resonance imaging compared to horses undergoing surgery. Equine Vet J. 2006;38(6):497‐501. 17124838 10.2746/042516406x156505

[26] Johnston GM , Eastment JK , Taylor PM , Wood JLN . Is isoflurane safer than halothane in equine anaesthesia? Results from a prospective multicentre randomised controlled trial. Equine Vet J. 2004;36(1):64‐71. 14756374 10.2746/0425164044864723

